# Dissecting the Catalytic Mechanism of *Trypanosoma brucei* Trypanothione Synthetase by Kinetic Analysis and Computational Modeling*

**DOI:** 10.1074/jbc.M113.483289

**Published:** 2013-06-28

**Authors:** Alejandro E. Leroux, Jurgen R. Haanstra, Barbara M. Bakker, R. Luise Krauth-Siegel

**Affiliations:** From the ‡ Biochemie-Zentrum der Universität Heidelberg, D-69120 Heidelberg, Germany,; § University of Groningen, University Medical Center Groningen, Department of Pediatrics, Center for Liver, Digestive, and Metabolic Diseases, Groningen NL-9713 GZ, The Netherlands, and; the ¶ Department of Molecular Cell Physiology, Faculty of Earth and Life Sciences, VU University, Amsterdam NL-1081 HV, The Netherlands

**Keywords:** Enzyme Kinetics, Glutathione, Mathematical Modeling, Thiol, Trypanosoma brucei, Glutathionylspermidine

## Abstract

In pathogenic trypanosomes, trypanothione synthetase (TryS) catalyzes the synthesis of both glutathionylspermidine (Gsp) and trypanothione (bis(glutathionyl)spermidine (T(SH)_2_)). Here we present a thorough kinetic analysis of *Trypanosoma brucei* TryS in a newly developed phosphate buffer system at pH 7.0 and 37 °C, mimicking the physiological environment of the enzyme in the cytosol of bloodstream parasites. Under these conditions, TryS displays *K_m_* values for GSH, ATP, spermidine, and Gsp of 34, 18, 687, and 32 μm, respectively, as well as *K_i_* values for GSH and T(SH)_2_ of 1 mm and 360 μm, respectively. As Gsp hydrolysis has a *K_m_* value of 5.6 mm, the *in vivo* amidase activity is probably negligible. To obtain deeper insight in the molecular mechanism of TryS, we have formulated alternative kinetic models, with elementary reaction steps represented by linear kinetic equations. The model parameters were fitted to the extensive matrix of steady-state data obtained for different substrate/product combinations under the *in vivo*-like conditions. The best model describes the full kinetic profile and is able to predict time course data that were not used for fitting. This system's biology approach to enzyme kinetics led us to conclude that (i) TryS follows a ter-reactant mechanism, (ii) the intermediate Gsp dissociates from the enzyme between the two catalytic steps, and (iii) T(SH)_2_ inhibits the enzyme by remaining bound at its product site and, as does the inhibitory GSH, by binding to the activated enzyme complex. The newly detected concerted substrate and product inhibition suggests that TryS activity is tightly regulated.

## Teaser

**Background:** Trypanothione synthetase catalyzes the conjugation of spermidine with two GSH molecules to form trypanothione.

**Results:** The kinetic parameters were measured under *in vivo*-like conditions. A mathematical model was developed describing the entire kinetic profile.

**Conclusion:** Trypanothione synthetase is affected by substrate and product inhibition.

**Significance:** The combined kinetic and modeling approaches provided a so far unprecedented insight in the mechanism of this parasite-specific enzyme.

## Introduction

Trypanosoma and *Leishmania* are the causative agents of tropical diseases such as African sleeping sickness and Nagana cattle disease (*Trypanosoma brucei* species), South-American Chagas disease (*Trypanosoma cruzi*), and the different forms of leishmaniasis. A common feature of these parasitic protozoa is their unusual thiol redox metabolism. They lack glutathione reductases and thioredoxin reductases but have a trypanothione/trypanothione reductase-based system instead. Trypanothione (*N*^1^,*N*^8^-bis(glutathionyl)spermidine (T(SH)_2_)^4^) is of pivotal importance for the viability and virulence of these parasites. The dithiol is the donor of reducing equivalents for the synthesis of DNA precursors by ribonucleotide reductase and the detoxification of hydrogen peroxide and lipid-derived hydroperoxides by different tryparedoxin peroxidases as well as the reduction of protein methionine sulfoxides (for a recent review, see Ref. 1).

T(SH)_2_ is synthesized from two molecules of GSH that are covalently bridged by a spermidine (Spd) in two ATP-dependent reactions. In the first step, glutathionylspermidine (Gsp) is formed, which is then combined with the second GSH to yield T(SH)_2_. In *Crithidia fasciculata*, an insect parasite widely studied as model trypanosomatid, two enzymes, a glutathionylspermidine synthetase (GspS) and trypanothione synthetase (TryS), are present (2, 3). A GspS, but not TryS, also occurs in *Escherichia coli*, the first organism for which a direct linkage of the GSH and Spd metabolism was described (4, 5). Gsp is formed when the bacteria enter the stationary phase and is broken down as soon as growth is restored. Both reactions are catalyzed by GspS, which is a bifunctional enzyme with Gsp synthetase and amidase activity (6).

**SCHEME 1:**
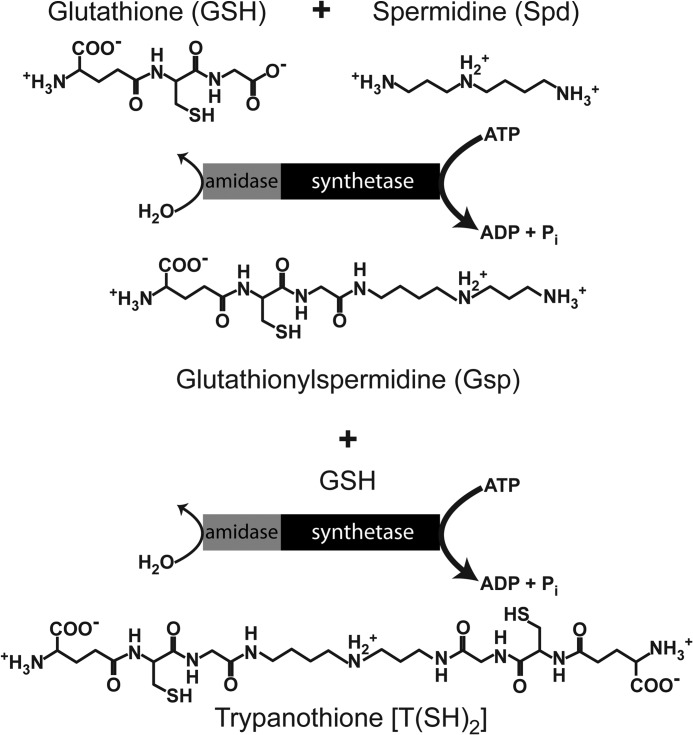


In contrast to *C. fasciculata*, the pathogenic *T. brucei* (7, 8), *T. cruzi* (9, 10), and *Leishmania major* (11) species employ TryS as a single enzyme to generate both Gsp and T(SH)_2_ (Scheme 1). *In vitro*, TryS also displays an amidase activity and in total can catalyze five different reactions: ATP hydrolysis and formation of Gsp and T(SH)_2_ as well as hydrolysis of the conjugates to regenerate GSH and Spd. The related GspS enzymes from *E. coli* and *C. fasciculata* catalyze the latter reaction at pH 7.5 and 25 °C, with a *K_m_* value of 0.9 and 0.5 mm and a *k*_cat_ of 2.1 and 0.38 s^−1^, respectively (2, 6). The amidase activity of the TryS enzymes appears to be comparably low. *T. cruzi* and *T. brucei* TryS hydrolyze Gsp at a rate that is less than 1% that of the synthetase activity, and cleavage of T(SH)_2_ is even slower (7, 9).

In the search for novel drug targets, pathways that are essential for parasite survival and are absent from the mammalian host are attractive starting points. Genetic and chemical approaches revealed that all proteins of the T(SH)_2_ system studied so far, such as γ-glutamylcysteine synthetase (12), TryS (8, 13), trypanothione reductase (14), and tryparedoxin (15) as well as the cytosolic 2-Cys-peroxiredoxin (16) and glutathione peroxidase-type enzymes (16–18) are essential for the viability of African trypanosomes. Several of them are currently investigated as potential drug targets (19). From both a metabolic point of view and putative druggability, TryS has been suggested to have the highest therapeutic potential of all enzymes of the T(SH)_2_ system (19, 20).

A final conclusion as to which protein plays a main role in the pathway control would need a reliable computational model based on knowledge of the kinetic parameters for all enzymes under identical conditions (21). Even more importantly, the kinetic data should be measured under conditions that resemble the milieu in which the pathway is active rather than at a non-physiological optimum (22). The importance of intracellular conditions for metabolic functions could recently be demonstrated; that is, the use of an “*in vivo*-like” assay medium yielded enzyme kinetic parameters that substantially improved a computational model of yeast glycolysis (23). Such data are not yet available for the parasite T(SH)_2_ metabolism. As in other cases, the proteins were characterized under conditions giving the highest activities for the individual enzyme studied. Thus, different buffer compositions, ionic strengths, and pH values were applied. For instance, the kinetic parameters of *T. brucei* TryS were measured in HEPPS buffer at pH 8.0 and 25 °C (7). Another characteristic of the intracellular milieu is the simultaneous presence of substrates and products, but product inhibition of TryS has not been investigated either.

Here we present a comprehensive analysis of *T. brucei* TryS under conditions that resemble the *in vivo* situation. We developed a phosphate-based buffer system at pH 7.0 that mimics the cytosol of bloodstream African trypanosome and is recommended for the comparative studies of all enzymes from the *T. brucei* cytosol. The complexity of the catalytic mechanism of TryS, including two catalytic cycles and inhibition by the substrate GSH as well as by the product T(SH)_2_, led us to build a mathematical model that allowed the incorporation of all kinetic data. The combination of measurements under *in vivo*-like conditions at 37 °C with computational modeling indeed provided us with interesting novel insights in the kinetic mechanism of TryS.

## EXPERIMENTAL PROCEDURES

### 

#### 

##### Materials

NADH was purchased from Biomol, monobromobimane was from Calbiochem, HEPES and Tris were from Roth, and Spd, GSH, ATP, phosphoenolpyruvate, rabbit pyruvate kinase, and bovine l-lactate dehydrogenase were from Sigma. T(SH)_2_ and trypanothione disulfide (TS_2_) were prepared enzymatically (24). Gsp was produced essentially by the same procedure, replacing TryS by *C. fasciculata* C79A GspS.^5^

##### Cloning, Overexpression, and Purification of Recombinant Tag-free TryS

The coding region of TryS was amplified from genomic DNA of the *T. brucei* 449 strain using *Pfu* DNA polymerase (Fermentas) (94 °C for 2 min, 94 °C for 30 s, 57 °C for 30 s, and 72 °C for 2 min for 25 cycles and 72 °C for 10 min). The forward primer (5′-CC ATG GGC ATG ACG AAG TCG-3′) contained an NcoI restriction site and the reverse primer (5′-GG TAC CTA CAT TTG AAT ACG TAC GGG A-3′) introduced an Acc65I site after the stop codon. The PCR product was ligated into the pET-blue1 vector (Novagen) and amplified in *E. coli* Nova Blue cells (Novagen). An NcoI site in the coding region was removed with the QuikChange® site-directed mutagenesis kit (Stratagene) with the primers TryS-mut-F (GTT AAT GAG GAT GCG CC*G* TGG GGA CAT GTC GCG) and TryS-mut-R (CGC GAC ATG TCC CCA *C*GG CGC ATC CTC ATT AAC) following the provider's instructions. After digestion with NcoI and Acc65I, the fragment was ligated into the pETtrx1b vector (kindly provided by Gunther Stier) and fully sequenced (GATC Biotech AG, Konstanz, Germany). *E. coli* Tuner(DE3) competent cells (Novagen) were transformed with the plasmid and grown in 1 liter of Terrific Broth medium containing 50 μg/ml kanamycin at 37 °C. At an OD_600_ of 0.6 the expression was induced by 0.3 mm isopropyl-β-d-thiogalactopyranoside. The cells were allowed to grow overnight at 25 °C, harvested, and resuspended in 30 ml of buffer A (50 mm Tris-HCl, 300 mm NaCl, pH 8.0) containing 150 nm pepstatin, 4 nm cystatin, 100 μm phenylmethylsulfonyl fluoride, 6 mg of lysozyme, and 0.6 mg of DNase A. The bacteria were disintegrated by sonication, and the clarified extract was loaded onto a nickel-nitrilotriacetic acid Superflow matrix (Qiagen) following an established procedure (25). The fusion protein was eluted with 30 mm imidazole in buffer A, concentrated on a 50-kDa Amicon filter (Millipore), and digested overnight at 4 °C with His-tagged tobacco etch virus protease (26). About 10 mg of tag-free TryS was collected from the run-through of a second nickel column. The purity of the protein was ≥95% according to SDS-PAGE. Recombinant TryS was stored at a concentration of 2 mg/ml at 4 °C. The protein concentration was determined by a Bradford assay using bovine serum albumin as standard. A 1 mg/ml solution of pure tag-free *T. brucei* TryS shows an *A*_280 nm_ of 1.9.

##### Kinetic Characterization of the Synthetase Activity

To allow comparison with published data (7), TryS was first studied at 25 °C and 37 °C in 100 mm K-HEPES, pH 8.0, supplemented with 5 mm dithiothreitol, 0.5 mm EDTA, and 10 mm MgCl_2_. To mimic the physiological environment of the enzyme, the activity was measured at 37 °C in 10 mm potassium phosphate, pH 7.0, containing with 15 mm NaCl, 85 mm KCl, and 10 mm MgCl_2_ (*in vivo*-like buffer system). The substrate stock solutions were prepared in the respective buffer and, if necessary, the pH was adjusted. The ATP stock solution consisted each of 100 mm ATP and MgCl_2_. The assays were performed in a final volume of 0.2 ml containing 0.2 mm NADH, 1 mm phosphoenolpyruvate, 0.4 units of pyruvate kinase, 0.4 units of lactate dehydrogenase, 0.2–0.8 μm TryS, and varying concentrations of ATP, GSH, and Spd or Gsp. The standard assay contained ATP, GSH, and Spd at fixed concentrations of 2.1, 0.1, and 20 mm, respectively. The assay mixtures were preincubated for 2 min before the reaction was started by the addition of Spd or Gsp and the absorption decrease at 340 nm was followed in a UV-visible V-650 spectrophotometer (Jasco). After each assay under the *in vivo*-like conditions, the pH of the reaction mixture was controlled to exclude significant changes. The apparent *K_m_* values and *k*_cat_ were determined by varying the respective substrate at fixed concentrations of the co-substrates (2.3 mm ATP, 1.0 mm GSH, and 8 mm Spd, or 0.5 mm Gsp). The concentrations ranged between 0.005 and 2.5 mm for ATP, 0.04 and 2.0 mm for GSH, 0.12 and 8.0 mm for Spd, and 0.01 and 0.50 mm for Gsp. The parameters were determined by non-linear regression; the experimental data were fitted to the specific function applying the Gauss-Newton algorithm (27). All data used for kinetic model construction were obtained under the *in vivo*-like conditions. Product inhibition of TryS was followed by varying one substrate at fixed concentrations of T(SH)_2_.

**FIGURE 1.**
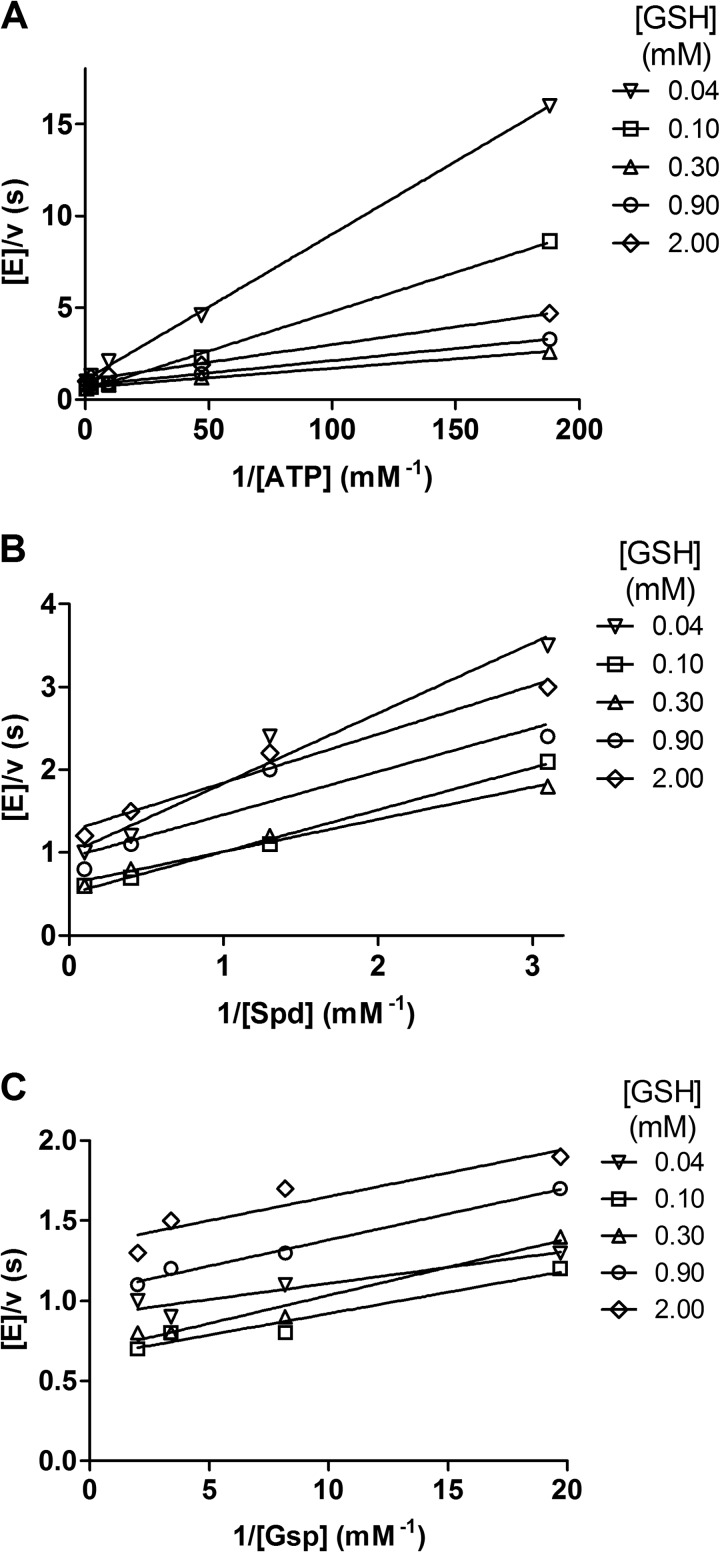
**Steady-state kinetic analysis of TryS.** The activities were measured in the *in vivo*-like buffer system varying the concentration of two substrates while keeping one constant at saturating concentrations. Shown are double reciprocal plots of the reactions with fixed concentrations: 8 mm Spd (*A*), 2.3 mm ATP (*B*), and 2.3 mm ATP (*C*).

**FIGURE 2.**
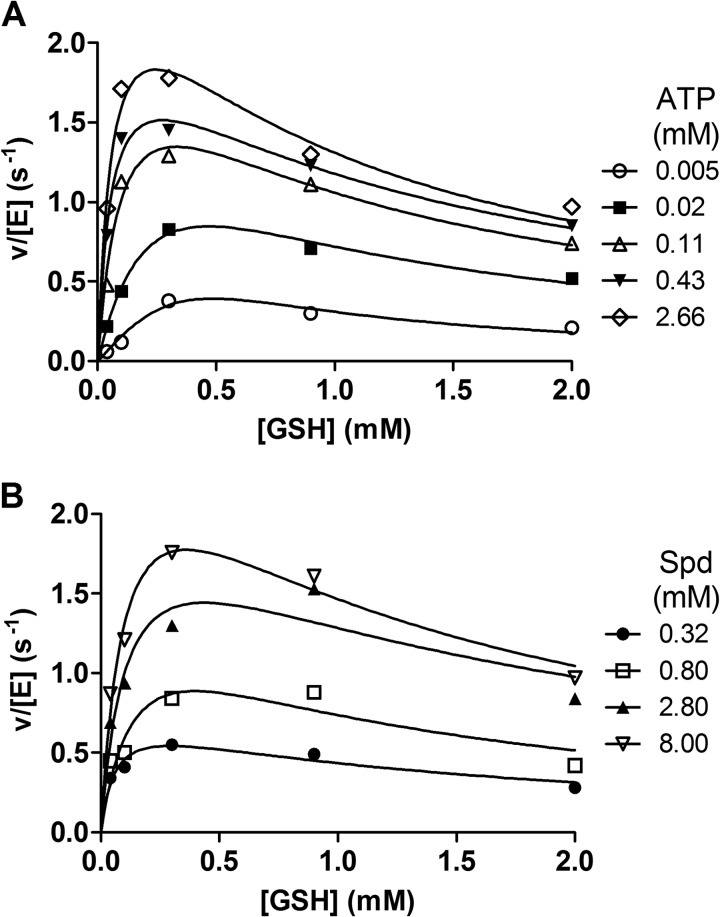
**Substrate inhibition of TryS by GSH.** The activity of TryS at variable concentrations of GSH was measured in the *in vivo*-like buffer system. The assays contained 8 mm Spd and different fixed ATP concentrations (*A*) and 2.3 mm ATP and different fixed Spd concentrations (*B*). Data were fitted to the high substrate inhibition equation as described under “Results.”

**FIGURE 3.**
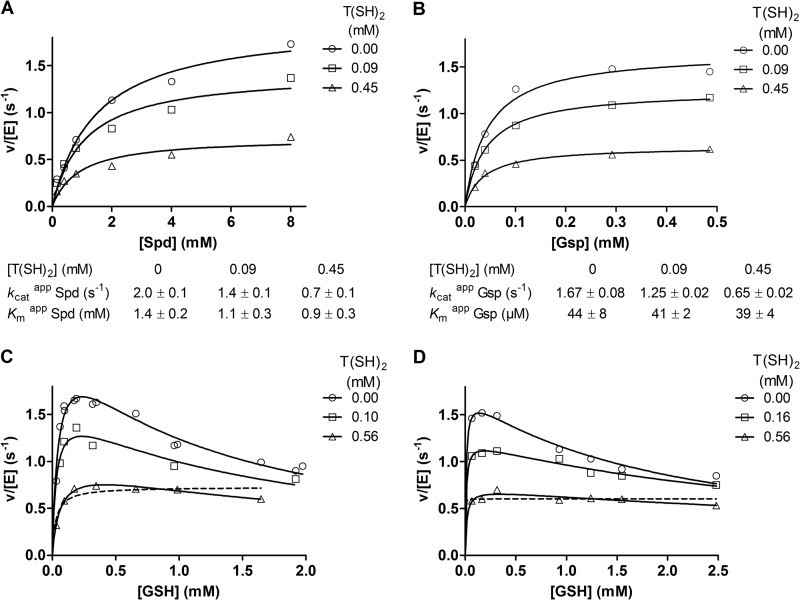
**Product inhibition of TryS by T(SH)_2_.** The assays were conducted in the *in vivo*-like buffer system. The reaction mixtures contained in the presence or absence of T(SH)_2_ 0.2 mm GSH, 2.3 mm ATP, and variable Spd concentrations (*A*), 0.2 mm GSH, 2.3 mm ATP, and variable Gsp concentrations (*B*), 8 mm Spd, 2.3 mm ATP, and variable GSH concentrations (*C*), and 0.52 mm Gsp, 2.3 mm ATP, and variable GSH concentrations (*D*). Data in *A* and *B* were fitted to the Michaelis-Menten equation. The kinetic constants derived are given *below the graphs*. Data in *C* and *D* were fitted to the high substrate inhibition equation (*solid lines*) and for the highest T(SH)_2_ concentration also to the Michaelis-Menten equation (*dashed line*).

To get the highest possible data density, we have chosen not to replicate each individual experiment but rather measure at many different substrate and product concentrations. Therefore, Figs. 1–3 show single measurements. The full dataset consists of 288 single measurements and is available at SysMO SEEK database. The internal consistency of the dataset is demonstrated by the smoothness of curves and the reproducible results obtained for occasional replicates.

##### HPLC Quantification of Gsp and T(SH)_2_

In 2 ml of the *in vivo*-like buffer, 170 nm TryS was mixed with 2.1 mm ATP and different concentrations of GSH and Spd as well as all components for the coupled enzymatic assay (see above), and NADH consumption was followed at 37 °C in the spectrophotometer. At different time points (during the first 2 min every 20 s and then at 4, 8, 15, and 24 min), a 100-μl aliquot was removed, and the reaction was immediately stopped by adding 10 μl of 100% ice-cold trichloroacetic acid. After centrifugation, the supernatant was extracted with diethyl ether. The thiols were derivatized with monobromobimane, and the samples were subjected to HPLC analysis as previously described (28). Standard solutions containing 5, 20, or 50 μm of each Gsp and T(SH)_2_ were prepared under identical conditions but omitting TryS.

##### ATPase and Amidase Activity of TryS

The ATPase activity was assessed using the synthetase assay lacking at least one of the other two substrates. The assays contained between 0.2 and 10 mm ATP and 2–10 μm TryS. The amidase activity was measured at 37 °C in 0.6 ml of the *in vivo*-like buffer containing 1.2 μm TryS and 0.1, 0.5, 2, or 8 mm Gsp. After 2, 5, 10, 30, and 60 min, a 100-μl aliquot was removed, and the reaction immediately stopped by adding 10 μl of 100% ice-cold trichloroacetic acid. The samples were diluted with 10% trichloroacetic acid to a maximum thiol concentration of 0.4 mm. The thiols were derivatized and analyzed by HPLC as described above. The initial velocities were calculated from the GSH produced within 5 and 10 min, respectively. To evaluate the effect of ATP on the amidase activity, reaction mixtures contained 1.2 μm TryS, 0.5 mm Gsp, and 0, 0.4, and 2.1 mm ATP, respectively.

##### Kinetic Model and Simulations

PySCeS (Python Simulator for Cellular Systems) open source modeling software (29) was used for initial model construction. Each elementary reaction step was described by reversible mass action kinetics, except the phosphorylation steps of GSH, which were modeled as irreversible. Alternative model versions were exported from PySCeS in sbml format to allow import into COPASI version 4.49.45 (30) for parameter estimation as well as steady-state and time course simulations. Constraints on the parameters were added to enforce microscopic reversibility in the central part of the model. For each face of the cubes (*i.e.* E-EA-EAB-EB-E) (Fig. 6) the product of the rate constants going clockwise should equal the product of the rate constants going counterclockwise (31). The concentrations in the model are in μm, and the time unit is in seconds. Fluxes of individual reactions within the model are in μm/s. For Spd as substrate, steady-state calculations were done at fixed concentrations of ATP, GSH, Spd, and T(SH)_2_. For Gsp, the procedure was the same except that the Gsp concentration was also fixed and that of Spd was zero. The model contains an irreversible reaction that consumes ADP, which mimics the coupling reaction in the enzymatic assay. In steady state, the rate of this reaction is equivalent to the overall ADP formation flux. For the steady-state simulations the total enzyme concentration, which is a conserved moiety, was set to 1 μm, and the calculated rates were normalized to enzyme concentration. Therefore, the overall ADP formation flux by the model is expressed in μm/s per 1 μm enzyme, leading to a unit of s^−1^. The time courses were simulated at 0.17 μm TryS, which was the enzyme concentration used in the corresponding experiment, with Gsp and T(SH)_2_ as variables.

##### Parameter Estimation

The kinetic constants in the model were fitted to the steady-state data obtained in the *in vivo*-like buffer. Parameter estimation was done in COPASI (30) by evolutionary programming (200 generations, population size of 20). The rate of the coupling reaction (which in steady state equals the ADP formation flux) was compared with the measured ADP production rate (in s^−1^), and the least sum of squares was the objective function to be minimized, *E*(*P*) = Σ*_i,j_*ω*_j_* × (x*_i,j_* − *y_i,j_*(*P*))^2^, in which *E* is the objective value, *P* is the tested parameter set, *x_i,j_* is a point in the dataset, and *y_i,j_*(*P*) is the corresponding simulated value. The indices *i* and *j* denote rows and columns in the dataset. The weight for each data column is given by ω*_j_*, which was set to a fixed value of one in all our calculations.

Parameter values to be fitted were allowed to vary between 1 × 10^−10^ and infinity. Because the Gsp concentration was a fixed parameter when it was the substrate but a variable intermediate with Spd as substrate, we needed subsequent rounds of parameter estimations using alternately the data on Spd or Gsp. The parameter estimation was started with a value of 100 for each parameter, and the Gsp data were fitted allowing all parameters to vary. Continuing from the resulting parameter set, the Spd data were fitted varying only the parameters of the Spd branch (see Fig. 6) with Gsp concentration as a variable. Next, the parameters of the Gsp branch were refitted to the Gsp data, and in a final round the parameters of the Spd branch were refitted to the Spd data. Additional rounds did not decrease the overall objective value (sum of least squares on Spd data and Gsp together).

##### Evaluation of Models

Alternative models with fitted parameters were evaluated first by comparing the objective value of the estimation between the different models. Models with the lowest objective value were further inspected by visual comparison of the model calculations with the experimental data for key behavior of the enzyme (*i.e.* how the model performs at different substrates ratios, the shape of the GSH inhibition curve, and inhibition by T(SH)_2_). The time-course data were never included in the parameter estimations but were used for independent validation.

## RESULTS

### 

#### 

##### Setup of a Buffer System That Mimics the Cytosol of Bloodstream T. brucei

Based on published data (32–34), a phosphate buffer system was established which mimics the cytosol of the cell and is composed of 100 mm potassium (32), 15 mm sodium (32), 10 mm magnesium, 120 mm chloride (32), 10 mm total phosphate and has a pH of 7.0 (33, 34). From the total magnesium content of *T. brucei* (35), a concentration of 6 mm magnesium in the cytosol of bloodstream cells could be estimated. This value fits to the 0.3–1.5 mm free magnesium reported for other cells taking into account that the total concentration is 10–20 times higher (36). In addition, polyamines can compete with magnesium for ATP binding (36). Thus, a magnesium concentration of 10 mm was selected, and the ATP stock solution was made of each 100 mm concentration of ATP and MgCl_2_ to ensure the presence of Mg-ATP throughout the ATP concentration range studied.

The total concentration of inorganic phosphate in *T. brucei* has been found to be 20–40 mm (37). The cytosolic level may be considerably lower because of the high phosphate concentration in the acidocalcisomes of the parasites. The activity of TryS was about 25% diminished in a 30 mm phosphate compared with 10 mm phosphate buffer, pH 7.0. This was not due to the higher ionic strength because the addition of 100 mm NaCl to the HEPES buffer, pH 8.0, did not affect the enzyme activity (data not shown). As different mammalian cells have been reported to have cytosolic phosphate concentrations between 1 and 10 mm (38–40), the phosphate concentration was finally set to 10 mm. The overall calculated ionic strength of this *in vivo*-like buffer is 200 mm, which corresponds to that in red blood cells (41). Macromolecular crowding agents (polyethylene glycol or BSA) mimicking the high intracellular protein concentration did not affect the enzyme activity significantly and were therefore not included in the assay medium.

##### Buffer Species, pH, and Temperature Strongly Affect the Kinetic Constants of TryS

TryS was analyzed in HEPES buffer at pH 8.0 and 7.0 and in the *in vivo*-like phosphate buffer, pH 7.0. Assays were conducted at 25 °C to allow comparison with published data and at 37 °C, reflecting the physiological environment of the parasite in the mammalian host. Hyperbolic kinetics were obtained when ATP, Spd, or Gsp was the variable substrate. In contrast, TryS displayed substrate inhibition when GSH was varied. In the latter case, the experimental data were fitted to Haldane's high substrate inhibition equation, *v* = *V*_max_/(1 + *K_m_*/*S* + *S*/*K_i_*), which is based on unproductive binding of GSH to the substituted enzyme (42). This yielded the *K_m_* and *K_i_* values for GSH as well as the *k*_cat_ depicted in Table 1. At varying ATP, Spd, or Gsp concentrations, the enzyme showed pronounced inhibition at GSH concentrations of ≥300 μm (see below and Fig. 2). Under these conditions, the calculated *k*_cat_ values were, therefore, lower than those given in Table 1 (see for instance Table 2).

**TABLE 1:** **Kinetic parameters of *T. brucei* TryS in different assay systems** The fixed substrate concentrations used in the HEPES system were 2.5 mm ATP, 1 mm GSH, and 20 mm Spd and in the *in vivo*-like phosphate buffer 2.5 mm ATP, 2 mm GSH, 8 mm Spd, and 0.5 mm Gsp. Inhibition of TryS by T(SH)_2_ *versus* Spd or Gsp was studied at fixed 0.2 mm GSH (for details see the legend of Fig. 3). The kinetic parameters, which are the means of at least two series of experiments ± S.E. were obtained as described under “Experimental Procedures.” ND, not determined.

Kinetic parameters	HEPPS, pH 8.0*^a^*	HEPES, pH 8.0		Phosphate, pH 7.0
	25 °C	25 °C	37 °C	37 °C
	μ*m*	μ*m*		μ*m*
*K*_*m*_^app^ Spd	38 ± 5	92 ± 25	139 ± 14	687 ± 90
*K*_*m*_^app^ Gsp	2.4 ± 0.2	12 ± 2	16 ± 3	32 ± 5

**With Spd as substrate**				
*K*_*m*_^app^ GSH	56 ± 11	32 ± 4	45 ± 3	69 ± 5
*K*_*i*_^app^ GSH	37 ± 7	143 ± 20	332 ± 22	849 ± 115
*K*_*m*_^app^ ATP	7.1 ± 0.4	6.6 ± 0.5	10 ± 2	18 ± 4
*k*_cat_ (s^−1^)	2.9 ± 0.4	1.8 ± 0.1	5.2 ± 0.2	2.8 ± 0.1
*K*_*i*_^app^ T(SH)_2_*^b^*	ND	ND	ND	228 ± 112
*K*_*i*_^app^ T(SH)_2_*^c^*	ND	ND	ND	377 ± 64

**With Gsp as substrate**				
*K*_*m*_^app^ GSH	ND	ND	ND	34 ± 12
*K*_*i*_^app^ GSH	ND	ND	ND	1085 ± 309
*K*_*m*_^app^ ATP	ND	ND	ND	12 ± 3
*k*_cat_ (s^−1^)	ND	ND	ND	2.1 ± 0.3
*K*_*i*_^app^ T(SH)_2_*^d^*	ND	ND	ND	223 ± 100
*K*_*i*_^app^ T(SH)_2_*^c^*	ND	ND	ND	346 ± 25

*^a^* Data are from Oza *et al.* (7), with 2 mm ATP, 0.1 mm GSH, and 10 mm Spd as fixed substrate concentrations.

*^b^* Apparent uncompetitive inhibitor constant *versus* the substrate Spd.

*^c^* Apparent uncompetitive inhibitor constant *versus* the substrate GSH.

*^d^* Apparent uncompetitive inhibitor constant *versus* the substrate Gsp.

**TABLE 2:** **Effect of buffer species, pH, and temperature on the kinetic parameters of TryS for Spd** The apparent *K_m_* values for Spd were determined at a fixed ATP and GSH concentration of 2.1 and 1 mm, respectively. The kinetic parameters, which are the means of at least two determinations ± S.E., were obtained as described under “Experimental Procedures.”

Buffer conditions	*K*_*m*_^app^	*k*_cat_^app^
	(μ*m*)	(*s*^−*1*^)
HEPES, pH 8.0 (25 °C)	92 ± 25	0.13 ± 0.01
HEPES, pH 7.0 (25 °C)	397 ± 72	0.15 ± 0.01
HEPES, pH 7.0 + 10 mm phosphate (25 °C)	431 ± 40	0.14 ± 0.01
Phosphate, pH 7.0 (25 °C)	545 ± 38	0.28 ± 0.01
Phosphate, pH 7.0 (37 °C)	766 ± 77	1.01 ± 0.05

The kinetic parameters of TryS obtained in HEPES, pH 8.0, at 25 °C were comparable with published data using HEPPS, pH 8.0 (7). The most obvious difference was the ratio between the *K_m_* and *K_i_* values for GSH (Table 1). Oza *et al.* (7) reported a *K_i_* value for GSH that was lower than the respective *K_m_* value. This was not the case in our assays in accordance with a later study by Torrie *et al.* (43). Changing the temperature from 25 °C to 37 °C resulted in slightly higher *K_m_* values for all substrates and shifted the *K_i_* value for GSH from 143 to 332 μm. As expected, the strongest effect was on *k*_cat_, which increased nearly 3-fold from 1.8 to 5.2 s^−1^.

For model construction, TryS was then thoroughly analyzed in the *in vivo*-like phosphate buffer at pH 7.0 and 37 °C. A pronounced difference in comparison to HEPES at pH 8.0 was the 3-fold higher *K_i_* value for GSH. Other major alterations were a rise of the *K_m_* value for Spd from 139 to 687 μm and the reduction of the *k*_cat_ from 5.2 to 2.8 s^−1^. To get a deeper insight in the underlying mechanism, the kinetic parameters for Spd were assessed in HEPES buffer at pH 8.0 and 7.0 in the presence and absence of 10 mm phosphate and compared with those obtained in the *in vivo*-like buffer (Table 2). This analysis revealed the pH shift as the main factor for the 5-fold difference in the *K_m_* value for Spd. The p*K_a_* values of the amino groups of Spd are 8.30, 9.90, and 10.66 (44), and lowering of the pH affects their ionization state. The higher *K_m_* value at pH 7.0 compared with pH 8.0 suggests that TryS preferably interacts with a less protonated form of Spd. A similar pH dependence has been described for *E. coli* GspS where changing the pH from 7.5 to 6.8 resulted in a 3-fold increase of the *K_m_* value for Spd (6). With Gsp as the third substrate, TryS showed a *k*_cat_ of 2.1 s^−1^, which is slightly lower than with Spd. Although not as pronounced as in the case of Spd, the *K_m_* value of Gsp also increased with decreasing pH. Taken together, pH, temperature, and buffer composition have a strong impact on the kinetic parameters of TryS.

##### Evaluation of the Catalytic Mechanism of TryS by Steady-state Kinetics

Using the *in vivo*-like buffer system, a matrix of kinetic data at various substrate and product concentrations was collected. At fixed Spd and variable GSH and ATP, the regression lines in the double reciprocal plot converged, suggesting the presence of a quaternary enzyme-substrate complex (Fig. 1*A*). In contrast, at fixed ATP and variable GSH and Spd or Gsp concentrations, the reciprocal plots revealed a reproducible but not clear picture (Fig. 1, *B* and *C*). The lines did not cut in a common point, and several of them appeared to be parallel, a pattern expected for a ping-pong mechanism. However, this is clearly not the case. In all three series of kinetics, independent of the nature of fixed and variable substrates, the lines did not follow the order expected when stepwise changing the GSH concentration. The activity of TryS increased from 40 to 100 and 300 μm GSH but then dropped when the concentration was further raised (Fig. 1) because GSH is not only a substrate but also an inhibitor of the enzyme (Table 1, see also the next section). The irregular pattern when Spd or Gsp was varied (Fig. 1, *B* and *C*) is most likely also due to the GSH inhibitory effect.

##### Mechanism of Inhibition of TryS by Its Substrate GSH

GSH acts as both substrate and inhibitor of TryS from several species (Table 1 and Refs. 3, 7, 9, 11, and 43). To get a deeper insight in the inhibition mechanism, the activity of TryS was followed at variable GSH and fixed saturating co-substrates concentrations (Fig. 2). The data obtained were fitted to the Haldane's high substrate inhibition equation (42) as well as to a more complex equation that takes into account a random-order binding of substrates to both the catalytically active and inactive enzyme and the putative generation of product from the inhibited ternary complex: *v* = (*V*_max1_+ *V*_max2_*S*/*K_i_*)/(1 + *K_m_*/*S* + *S*/*K_i_* +1/*K_i_K*_1_) (45). In all cases fitting of the kinetic data to the latter equation yielded zero and extremely high values, respectively, for *V*_max2_ (activity of the enzyme with GSH bound at the catalytic and inhibitory sites) and *K*_1_ (data not shown). This reduced the equation exactly to the simpler one and strongly suggests that TryS with GSH bound at an inhibitory site is trapped in an unproductive complex. When Gsp served as co-substrate, the calculated apparent *K_i_* value for GSH was about 1 mm, comparable with that obtained in the presence of Spd (Table 1).

##### Product Inhibition of TryS by T(SH)_2_

At fixed concentrations of ATP and GSH, the presence of T(SH)_2_ lowered the apparent *k*_cat_ and slightly affected the apparent *K_m_* value for Spd (Fig. 3, *A* and *B*). Thus the data were fitted to both non-competitive and uncompetitive inhibition equations. The separate analysis of each individual experiment did not allow a differentiation between both inhibitor types, and a further analysis by mathematical modeling was required (see below). When GSH was varied at fixed concentrations of the other substrates, T(SH)_2_ significantly affected the apparent *k*_cat_. Fitting the data to an uncompetitive inhibitor equation yielded a *K_i_* value for T(SH)_2_ of about 360 μm, independently if Spd or Gsp was the third substrate (Table 1). Interestingly, at high T(SH)_2_ concentrations the velocity curves for varying GSH adopted a nearly normal hyperbolic shape (Fig. 3, *C* and *D*). With Spd as third substrate, the calculated *K_i_* value for GSH was raised from 0.9 mm in the absence to 1.7 and 4.5 mm in the presence of 100 and 560 μm T(SH)_2_, respectively. With Gsp as substrate, the *K_i_* value for GSH was raised from 1.1 mm to 2.2 and 4.9 mm when the assay contained 160 and 560 μm T(SH)_2_, respectively. Thus, T(SH)_2_ seems to affect GSH binding at its inhibitory site.

##### Time Course of Gsp and T(SH)_2_ Production

**FIGURE 4.**
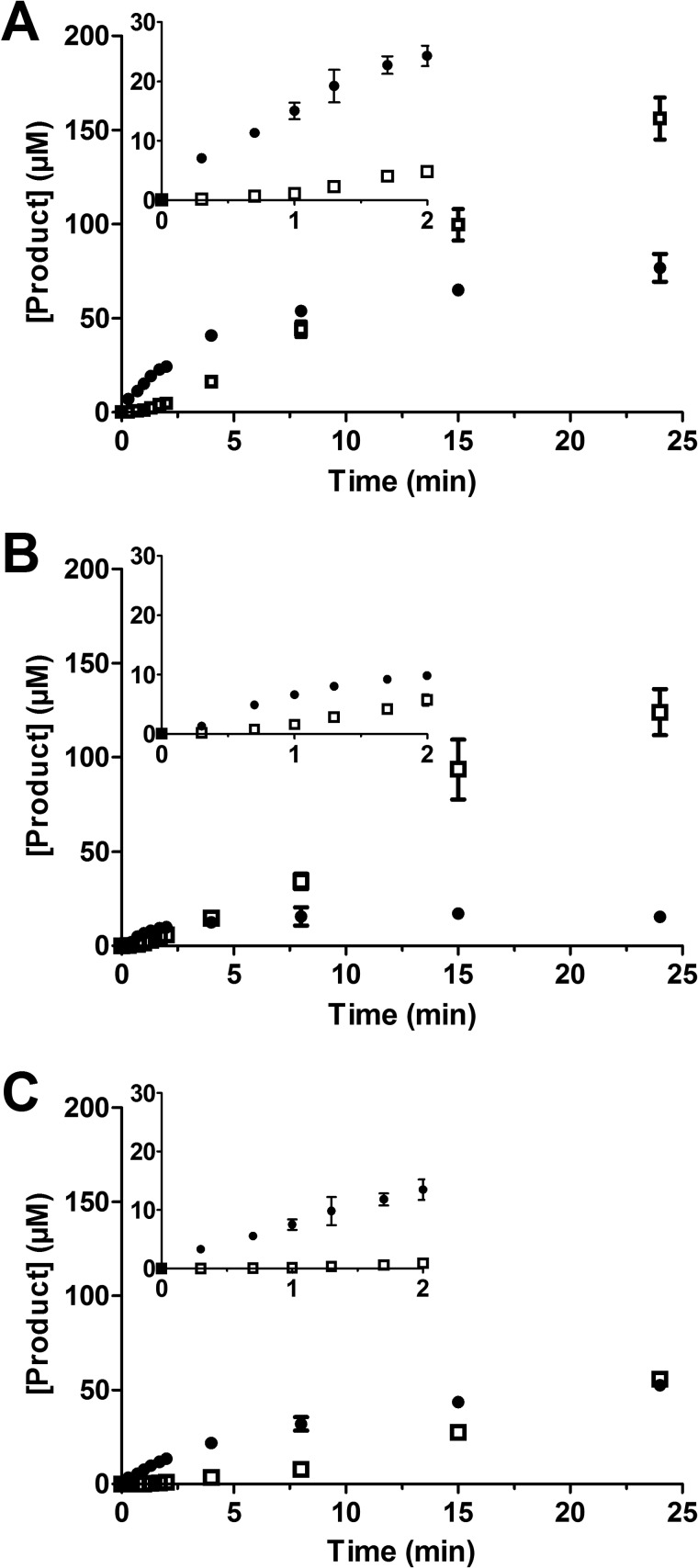
**Time-dependent production of Gsp and T(SH)_2_.** The reaction mixtures contained 2.1 mm ATP and 170 nm TryS as well as 0.33 mm GSH and 8.0 mm Spd (*A*), 0.33 mm GSH and 1.6 mm Spd (*B*), and 2.0 mm GSH and 8.0 mm Spd (*C*). After different time points, the Gsp (*filled circles*) and T(SH)_2_ (*open squares*) formed were quantified by HPLC analysis as outlined under “Experimental Procedures.” The average with S.D. of three independent experiments is shown. The *insets* provide an enlargement of the first 2 min.

The activity of TryS in the presence of different concentrations of GSH and Spd was followed spectrophotometrically. At different times, an aliquot was removed, and Gsp and T(SH)_2_ were quantified by HPLC analysis (Fig. 4). The theoretical NADH consumption was calculated from the sum of Gsp and T(SH)_2_ formed. Plotting these theoretical values against the measured NADH consumption yielded slopes between 0.8 and 1.15 and, thus, a good correlation between ATP turnover and Gsp and T(SH)_2_ production.

Independently of the substrate combinations, the synthesis of T(SH)_2_ started with a delay when compared with Gsp formation. During the first minute, T(SH)_2_ was practically undetectable but increased constantly as soon as Gsp had reached a steady-state level of 60 μm (in the presence of 8 mm Spd) or 15 μm (at 1.6 mm Spd) (Fig. 4, *A* and *B*). The dependence of the Gsp steady-state level on the Spd concentration is most likely due to the competition between both polyamine substrates for enzyme binding. Production of both Gsp and T(SH)_2_ was delayed when the reaction mixture contained 2 mm GSH instead of 0.3 mm GSH (Fig. 4*C*), in agreement with the very similar apparent *K_i_* value for GSH obtained in the presence of either substrate (Table 1). With Spd as substrate, NADH consumption mirrors the total ATP turnover for the production of Gsp and T(SH)_2_ in the two consecutive steps. The ATP consumption flux reaches steady state within 1 min after starting the assay, but the HPLC analysis showed that this reflects a changing distribution of Gsp and T(SH)_2_ produced.

##### TryS Has Negligible ATPase Activity

At concentrations between 0.8 and 4 mm ATP, a minute and constant ATPase activity of < 0.01 s^−1^ was observed that corresponded to less than 1% of the synthetase activity. The addition of either 3 mm GSH or 8 mm Spd did not significantly affect the reaction rate. In the presence of 0.6 mm Gsp, the consumption of ATP increased 25-fold. This does, however, not reflect an increased ATPase activity but is caused by the amidase activity of the enzyme (see the next section). The high enzyme concentration required for detecting any ATPase activity results in the hydrolysis of Gsp. In the presence of ATP, the GSH and Spd generated by this reaction fuel the synthetase reaction. Indeed, preincubation of TryS with Gsp resulted in a rapid increase in ATP consumption. Thus, the genuine ATPase activity of TryS is negligible and independent of the co-substrates.

##### The Amidase Activity of TryS under in Vivo Conditions Is Low

**FIGURE 5.**
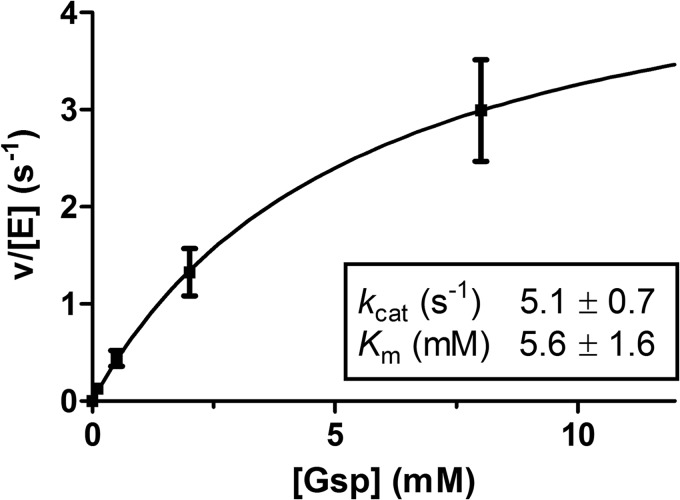
**Hydrolysis of Gsp by TryS.** The Gsp hydrolysis in the *in vivo*-like buffer system was followed by HPLC analysis. The reaction mixtures contained 1.2 μm TryS and 0.1, 0.5, 2, and 7 mm Gsp. The amidase activity as function of the Gsp concentration was fitted to a hyperbolic kinetic. The average with S.D. of at least three independent experiments is shown.

The ability of TryS to catalyze the hydrolysis of Gsp was studied at Gsp concentrations between 100 μm and 8 mm (Fig. 5). At different time points, the reaction was stopped and GSH and Gsp was quantified by HPLC analysis. GSH formation correlated with the consumption of Gsp, the sum of GSH and residual Gsp corresponding to 90–110% of the initial Gsp. Irrespective of the starting Gsp concentration, GSH production was linear during the first 10 min, allowing calculation of initial velocities from the 5- and 10-min time points. The amidase activity was fitted to a Michaelis-Menten-type kinetic (Fig. 5), which yielded a *k*_cat_ and *K_m_* values of 5.1 ± 0.7 s^−1^ and 5.6 ± 1.6 mm, respectively. Although the *k*_cat_ of the amidase activity was twice that of the synthetase activity (see Table 1), the *K_m_* value for Gsp was almost 200 times higher (5.6 mm compared with 0.03 mm). Thus, the amidase activity shows a catalytic efficiency of 9.1 × 10^2^ m^−1^s^−1^ that is nearly 2 orders of magnitude lower than that of synthetase reaction (6.6 × 10^4^ m^−1^s^−1^).

For *E. coli* GspS (46), it has been reported that the synthetase substrates, and especially Mg-ATP, highly activate the amidase activity toward an artificial substrate. The amidase activity of *T. brucei* TryS was studied at a fixed concentration of 0.5 mm Gsp. The presence of 0.4 or 2.1 mm ATP stopped GSH accumulation and triggered the synthesis of T(SH)_2_ (data not shown). Thus, ATP clearly did not stimulate the amidase activity. As the maximum Gsp concentration used in our kinetic analysis of TryS was 0.6 mm and all assays contained ATP, the amidase activity has not been taken into account in the kinetic model.

##### The Kinetic Model

**FIGURE 6.**
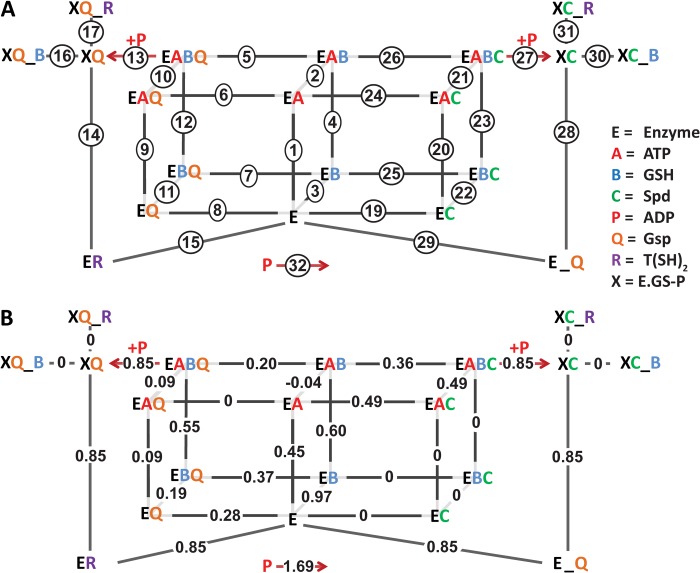
**Model of the ter-reactant mechanism of TryS catalysis.** *A*, all reactions in the model consist of reversible mass action kinetics, except for those that generate ADP (reactions *13* and *27*). The model includes a “coupling reaction” (reaction *32*) that consumes the ADP formed in one (on Gsp) or two (on Spd) reactions. Compounds connected to the enzyme complex with an underscore (_) represent a complex that cannot proceed in the catalytic cycle, *e.g.* Gsp that is formed by the enzyme as product (*E_Q*) is in a wrong orientation in the substrate cavity for the second reaction. The activated enzyme complexes (XC or XQ) are the species formed after ATP and GSH reacted to generate a phosphorylated GSH intermediate and ADP. *B*, the *numbers* correspond to the steady-state flux values (μm/s) obtained during a simulation using 1 μm TryS and physiological substrate concentrations (2.3 mm ATP, 0.3 mm GSH, and 8 mm Spd). Positive values represent fluxes in the direction of product formation.

A kinetic model consisting of linear mass-action equations for all binding and reaction steps was constructed for the synthetase activity. To compare putative mechanisms, we did parameter estimations for different model versions describing alternative mechanisms. The final model, which was in best agreement with the data, had an objective value of 3.40 (μmol/s)^2^ and is shown in Fig. 6.

##### Binding of Substrates to the Enzyme in the Model

In the final model all three substrates (ATP, GSH, and either Spd or Gsp) bind to *T. brucei* TryS before phosphorylation of GSH by ATP occurs. This is in contrast to the mechanism proposed for the *Crithidia* enzyme (3) but in agreement with our steady-state kinetic analysis that discarded a ping-pong mechanism. Also, in a ping-pong mechanism, binding of Spd or Gsp after the phosphorylation step, would render the *K_m_* values for ATP and GSH independent of whether Spd or Gsp is used as the third substrate. However, our data yielded a *K_m_* value for GSH that was 2-fold higher on Spd than on Gsp (Table 1). Similar differences were observed for *C. fasciculata* and *T. cruzi* TrySs (3, 9). Thus, in our model, Spd or Gsp needs to bind before GSH becomes phosphorylated. Considering the lack of experimental evidence for a specifically ordered substrate binding, we did not impose any predefined order.

TryS produces Gsp as an intermediate in the reactions from Spd to T(SH)_2_ and catalyzes the reaction from Gsp to T(SH)_2_ with Gsp as the direct substrate (Scheme 1). In the HPLC experiments using Spd as substrate (Fig. 4), Gsp accumulated far above the enzyme concentration of 0.17 μm and must, therefore, be released from the enzyme. Hence the selected topology of our kinetic model does not allow the direct production of T(SH)_2_ from Spd; both Spd and Gsp can compete for binding to the enzyme complexes. Structural information (47, 48) from *L. major* TryS indicates that the Gsp produced from Spd has to change orientation in the active site for further catalysis. Accordingly, our kinetic model clearly differentiates between Gsp bound as product of the first reaction (E_Q) and as substrate of the second one (*EQ*; Fig. 6*A*).

##### Incorporating Inhibition by GSH and T(SH)_2_ in the Model

TryS is inhibited by the substrate GSH and the product T(SH)_2_. We first scrutinized the experimental data to find out how GSH inhibition is affected by the presence of other substrates. For this purpose the activity profiles with GSH as varied substrate (Fig. 2) were plotted for different concentrations of ATP, Spd, and Gsp. The percentage of inhibition at 2 mm GSH relative to the maximum activity in the curve was calculated. Inhibition by GSH was either independent of the other substrates or even enhanced at increasing substrate concentration (data not shown). Furthermore, the product T(SH)_2_ inhibited the enzyme without significantly affecting the apparent *K_m_* values for Spd or Gsp (Fig. 3, *A* and *B*) which excluded competition between T(SH)_2_ and Spd or Gsp. Instead T(SH)_2_ appears to interfere with the binding of GSH at the inhibitory site (Fig. 3, *C* and *D*). This prompted us to place the inhibitory GSH and T(SH)_2_ on the same enzyme-substrate complex. Because the GSH inhibition was not competitive *versus* ATP, Gsp, and Spd and seemed to be even stimulated by them, we included the inhibitory binding after all substrates had been bound to the activated enzyme (*XC* or *XQ*; Fig. 6*A*), resembling an uncompetitive mechanism. This yielded a very good fit throughout the data with this model (objective value, 3.40 (μmol/s)^2^). Alternative options, allowing either only the inhibitory GSH or both GSH and T(SH)_2_ to bind to the quaternary complexes (EABC/EABQ) yielded objective values of 10.7 and >100 (μmol/s)^2^, respectively. Further proof that these positions are not in agreement with the data came from a scheme that combined both options, *i.e.* binding of the inhibitors before and after phosphorylation (to EABC/EABQ as well as XC/XQ). When fitting the data to this model, the forward constants for binding GSH or T(SH)_2_ to the quaternary complexes became extremely low in the parameter estimation. Thus, these options were removed from the final model.

Because the steady-state data did not allow a distinction between uncompetitive or non-competitive inhibition mechanisms, we also made a model that included specific inhibitory binding of GSH and T(SH)_2_ to the free enzyme in addition to binding to the activated enzyme. The parameter estimation led to a marginally lower objective value of 3.12 (μmol/s)^2^ compared with 3.40 (μmol/s)^2^ for the final model (Fig. 6*A*). However, as the dissociation constants (reverse constant/forward constant) for GSH and T(SH)_2_ at the free enzyme were more than two orders of magnitude higher than at the activated enzyme complex (not shown), we concluded that inhibitory binding of GSH and T(SH)_2_ to the free enzyme was of minor physiological importance, and thus, this option was excluded from the final model.

##### Simulations with the Parameterized Model

**FIGURE 7.**
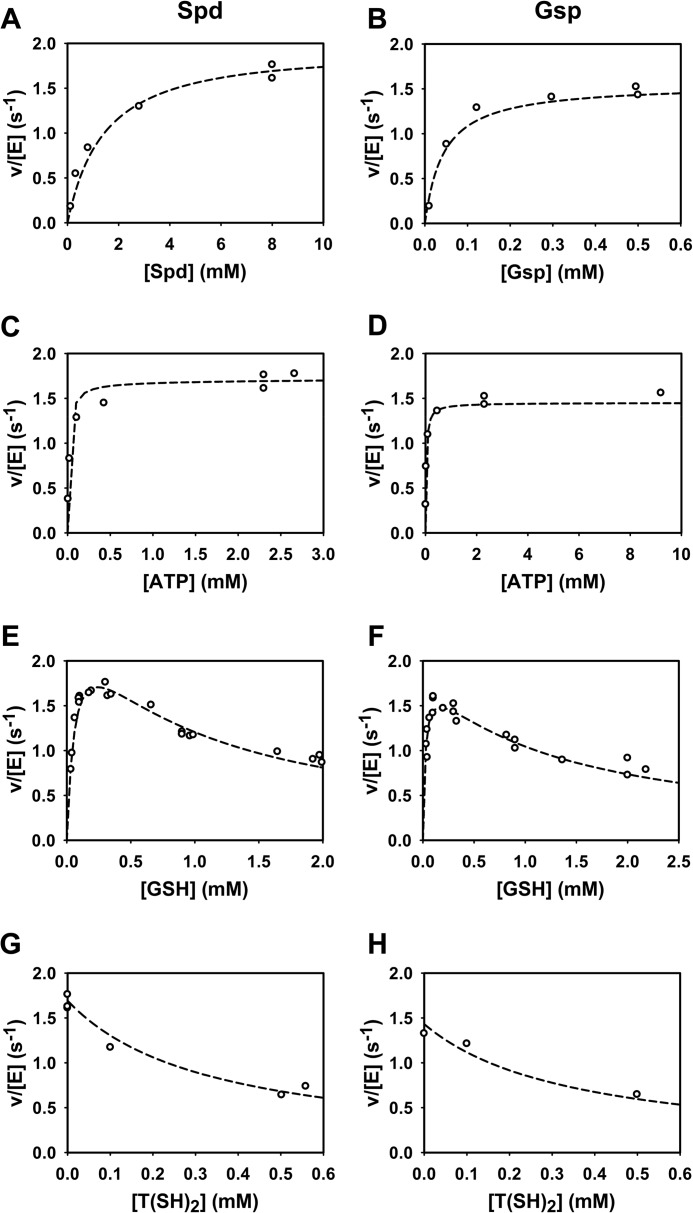
**Comparison of the model simulations with the experimental data of TryS.** Kinetic profiles of various reactions were measured with Spd (*left column*) and Gsp (*right column*) as substrates. The reaction mixtures contained fixed concentrations of 2.3 mm ATP and 0.3 mm GSH (*A* and *B*), 0.3 mm GSH and 8 mm Spd (*C*), 0.3 mm GSH and 0.5 mm Gsp (*D*), 2.3 mm ATP and 8 mm Spd (*E*), 2.3 mm ATP and 0.5 mm Gsp (*F*), 2.3 mm ATP, 0.3 mm GSH, and 8 mm Spd (*G*), 2.3 mm ATP, 0.3 mm GSH, and 0.5 mm Gsp (*H*). *Dashed lines* are model simulations, and *open circles* are experimental data (see Figs. 1, 2, and 3).

**FIGURE 8.**
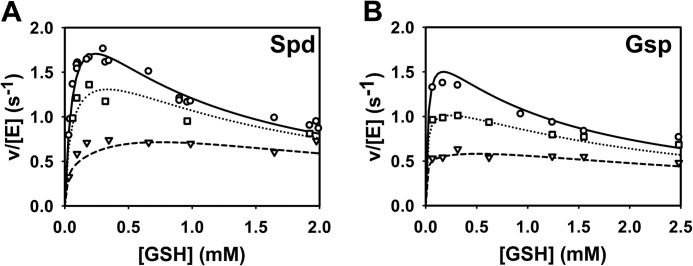
**Simulation of the T(SH)_2_ inhibition of TryS.** The activities of TryS measured at variable GSH and T(SH)_2_ concentrations at fixed concentrations of 2.3 mm ATP and 8 mm Spd (*A*) or 0.61 mm Gsp (*B*) (*symbols*) (see Fig. 3, *C* and *D*) are depicted together with the model simulation (*lines*). The reactions contained 0 mm (○, *solid line*), 0.1 mm (□, *dotted line*), and 0.56 mm T(SH)_2_ (▿, *dashed line*) (*A*) and 0 mm (○, *solid line*), 0.16 mm (□, *dotted line*), and 0.56 mm T(SH)_2_ (▿, *dashed line*) (*B*).

The final parameterized model (see Table 3 for the values of all constants) was used to simulate the experiments to which they were fitted. Key kinetics of the TryS model are shown in Fig. 7. The complete comparison of model results with all experimental data is available through the SysMO SEEK database and JWS online as well as from the authors upon request. The model simulations accurately describe the enzyme activity at variable substrate concentrations (Fig. 7, *A–D*). Both in the model and in the experimental data the maximum activity was slightly lower on Gsp than on Spd. The inhibition of TryS by T(SH)_2_ and GSH was quantitatively reproduced by the model (Fig. 7, *E–H*) as well as the shift of the GSH kinetic profile from a substrate inhibition curve to a more hyperbolic one in the presence of high T(SH)_2_ concentrations (Fig. 8).

**TABLE 3:** **Rate constants of the parameterized final model** The values were obtained through parameter estimation as described under “Experimental Procedures.” Constants with the suffix “a” describe the reverse reaction (the forward direction is in the direction of product formation). For example, the rate of reaction 1 (Fig. 6*A*) equals k1 × [E] × [A] − k1a × [EA]. Reaction 18 corresponded to the formation of an inhibitory enzyme/T(SH)_2_ complex in a previous model. As inclusion of this reaction did not improve the model (see “Results”), it was removed.

Parameter	Value	Unit	Parameter	Value	Unit
*k*_1_	13.5	μm^−1^s^−1^	*k*_16_*_a_*	2199	s^−1^
*k*_1_*_a_*	134.29	s^−1^	k_17_	48.53	μm^−1^s^−1^
*k*_2_	6317	μm^−1^s^−1^	*k*_17_*_a_*	82.5	s^−1^
*k*_2_*_a_*	42.45	s^−1^	*k*_19_	1.740 × 10^−2^	μm^−1^s^−1^
*k*_3_	223.4	μm^−1^s^−1^	*k*_19_*_a_*	868.3	s^−1^
*k*_3_*_a_*	286.7	s^−1^	*k*_20_	2.005 × 10^−6^	μm^−1^s^−1^
*k*_4_	762.3	μm^−1^s^−1^	*k*_20_*_a_*	1 × 10^−10^	s^−1^
*k*_4_*_a_*	39.72	s^−1^	*k*_21_	94.60	μm^−1^s^−1^
*k*_5_	22.92	μm^−1^s^−1^	*k*_21_*_a_*	5.663 × 10^4^	s^−1^
*k*_5_*_a_*	1465	s^−1^	*k*_22_	897.7	μm^−1^s^−1^
*k*_6_	6.497 × 10^−9^	μm^−1^s^−1^	*k*_22_*_a_*	26.8	s^−1^
*k*_6_*_a_*	1 × 10^−10^	s^−1^	*k*_23_	1 × 10^−10^	μm^−1^s^−1^
*k*_7_	7067	μm^−1^s^−1^	*k*_23_*_a_*	1 × 10^−10^	s^−1^
*k*_7_*_a_*	530.5	s^−1^	*k*_24_	5.468 × 10^4^	μm^−1^s^−1^
*k*_8_	125.3	μm^−1^s^−1^	*k*_24_*_a_*	1.368 × 10^4^	s^−1^
*k*_8_*_a_*	237.5	s^−1^	*k*_25_	8.612 × 10^−14^*^a^*	μm^−1^s^−1^
*k*_9_	369.7	μm^−1^s^−1^	*k*_25_*_a_*	1 × 10^−10^	s^−1^
*k*_9_*_a_*	29.88	s^−1^	*k*_26_	4.750 × 10^−2^	μm^−1^s^−1^
*k*_10_	261.2	μm^−1^s^−1^	*k*_26_*_a_*	1058	s^−1^
*k*_10_*_a_*	7289	s^−1^	*k*_27_	15.18	s^−1^
*k*_11_	6210	μm^−1^s^−1^	*k*_28_	455.5	s^−1^
*k*_11_*_a_*	315.7	s^−1^	*k*_28_*_a_*	290.4	s^−1^
*k*_12_	30.0	μm^−1^s^−1^	*k*_29_	3.025 × 10^4^	s^−1^
*k*_12_*_a_*	1331	s^−1^	*k*_29_a	200.1	μm^−1^s^−1^
*k*_13_	2.279	s^−1^	*k*_30_	234.2	μm^−1^s^−1^
*k*_14_	543.5	s^−1^	*k*_30_*_a_*	1219	s^−1^
*k*_14_*_a_*	1 × 10^−10^	s^−1^	*k*_31_	1 × 10^−10^	μm^−1^s^−1^
*k*_15_	34.93	s^−1^	*k*_31_*_a_*	420.6	s^−1^
*k*_15_*_a_*	4942	μm^−1^s^−1^	*k*_32_	1.425 × 10^4^	s^−1^
*k*_16_	488.9	μm^−1^s^−1^			

*^a^* Value lower than 1 × 10^−10^ but possible, as this constant was calculated from an assignment rule (see “Experimental Procedures”).

**FIGURE 9.**
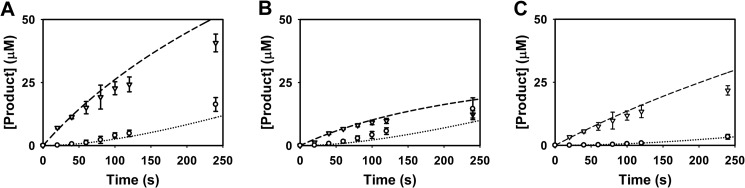
**Model simulation of the time-dependent production of Gsp and T(SH)_2_.** The measured (*symbols*) and simulated (*lines*) time-dependent production of Gsp (▿, *dashed line*) and T(SH)_2_ (○, *dotted line*) are depicted. The time frame shown corresponds to that used in the photometric assays. The experimental assays and simulations contained 0.17 μm TryS and 2.1 mm ATP as well as 0.33 mm GSH and 8.0 mm Spd (*A*), 0.33 mm GSH and 1.6 mm Spd (*B*), and 2.0 mm GSH and 8.0 mm Spd (*C*).

For an independent validation, the model was compared with the time course data of Gsp and T(SH)_2_ (Fig. 4) that had not been used for model fitting. In agreement with the experimental data, the model accumulated Gsp far above the enzyme concentration, reaching a steady-state level that was dependent on the Spd concentration (Fig. 9, *A* and *B*). Moreover, T(SH)_2_ formation exhibited a lag phase and was largely delayed in the presence of 2 mm GSH (Fig. 9*C*). Taken together, the simulations showed that the current mathematical model is able to qualitatively and quantitatively describe the TryS kinetic profile. However, there are some slight quantitative discrepancies, *e.g.* at the lower GSH concentrations in the presence of 0.56 mm T(SH)_2_ (Fig. 8*A*) and at the later time points in the time simulations (Fig. 9).

##### Steady-state Fluxes in the Model Show That Substrate Binding Is Partially Ordered

If the binding of the substrates was completely random, the binding constants of one substrate would not depend on whether other substrates are already bound to the enzyme. Although our model topology allows complete random, strictly ordered, or partially ordered (all constants can have unique values irrespective of the enzyme complex to which a substrate binds), the parameter estimation resulted in a final model where multiple orders of substrate binding exist in steady state (Fig. 6*B* and for more details see below). We have explicitly tested if a true random mechanism could accommodate the steady-state data. For this purpose we made a model with a single kinetic constant for the binding of a substrate irrespective of the enzyme-complex to which it binds. Although this model yielded good fits for either the Spd or the Gsp data alone, we could not find a parameter set that fitted both the Spd and Gsp data in a single model. The best fit had an overall objective value of 6.15 (μmol/s)^2^, higher than that of the final model (3.40 (μmol/s)^2^).

For analysis of the substrate binding order(s) we compared the steady-state fluxes along different binding paths, at near-physiological concentrations of the substrates. Fig. 6*B* provides the steady-state fluxes of each binding event in the presence of 8 mm Spd, 2.3 mm ATP and 0.3 mm GSH. The fluxes are distributed among alternative binding paths, yet certain paths carry a much smaller flux than others. Interestingly, Spd does not bind as first substrate but only after at least ATP has been bound. Moreover, although the model allows Gsp to bind as first substrate, there is a higher flux of binding after GSH. These results are in line with molecular dynamic simulations by Koch *et al.* (48), which indicate that Gsp and Spd binding is strongly influenced by the occupancy of the ATP or GSH binding sites.

##### How GSH and T(SH)_2_ Inhibit the Enzyme in the Model

Inhibition of the enzyme by GSH and T(SH)_2_ can be largely understood by looking at the dissociation constants *K_D_* (reverse constant/forward constant for a reaction; Table 3). Inhibitory binding of GSH and T(SH)_2_ results in dead-end complexes that are not part of the catalytic cycle (Fig. 6), and hence these binding events are in thermodynamic equilibrium when the enzyme works at steady state. The *K_D_* values for GSH as substrate depend on the complex to which it binds (1.3 μm for the free enzyme, 0.051 μm for EQ, and 599 μm for EAC), which is an additional demonstration of the non-random binding order. The *K_D_* values for GSH inhibition of the activated complexes (5.2 μm for XC and 4.2 μm for XQ) are similar, which suggests a binding site that becomes accessible in the activated enzyme irrespective of whether Spd or Gsp is bound.

The *K_D_* value for T(SH)_2_ binding to XQ is slightly lower (1.7 μm) than the respective value for GSH (4.5 μm), reflecting competition between GSH and T(SH)_2_ for inhibitory binding to the activated enzyme complex. Strikingly, the *K_D_* value for T(SH)_2_ binding to XC is very high. Apparently, inhibition at XQ is sufficient to explain the inhibition by T(SH)_2_ and its competition with GSH. From a structural point of view we cannot envision why T(SH)_2_ should only bind to one activated complex and not to the other. Imposing identical constants for binding of T(SH)_2_ to XC and XQ only slightly increased the objective value for this model (3.71 (μmol/s)^2^). Thus, we have insufficient data to quantify the affinity of T(SH)_2_ to XC.

The very low *K_D_* value for the complex of T(SH)_2_ with the free enzyme (7.1 nm) indicates that there is strong binding at the T(SH)_2_ production site. Indeed, at 0.56 mm T(SH)_2_ and 0.3 mm GSH, the ER complex composed 44% of the total enzyme pool in steady state, and 19% of enzyme was sequestered as XQ_R. The contribution of the latter complex is, however, essential, as removal of T(SH)_2_ binding to both activated enzyme species led to a model with poor performance in the parameter estimation (objective value 22.4 (μmol/s)^2^).

The distribution over the inhibitory complexes XC_B, XQ_B, XQ_R, and ER at different GSH concentrations gives insight in the competition between GSH and T(SH)_2_ inhibition. At 0.56 mm T(SH)_2_ and 0.3 mm GSH, as compared with GSH only, the fraction of enzyme in XC_B and XQ_B decreased from 10 to 3% in favor of ER (44%) and XQ_R (19%). In the absence of T(SH)_2_, increasing the concentration of GSH from 0.3 to 2 mm sequestered the enzyme in XC_B and XQ_B (each ∼35%). In the presence of 2 mm GSH and 0.56 mm T(SH)_2_, ∼50% of the enzyme were sequestered in XC_B and XQ_B together and 27% of the enzyme in XQ_R (17%) and ER (10%). Although the enzyme fraction that is sequestered by GSH decreases in the presence of T(SH)_2_, the total percentage of inhibitory complexes slightly increases when the product is added (from 70 to 77%). In conclusion, T(SH)_2_ inhibits TryS by remaining bound at its product site and, as it does the inhibitory GSH, by binding to the activated enzyme complex.

## DISCUSSION

Standard kinetic characterizations are usually done under conditions optimized to get the maximum activity of the particular enzyme. We show that the kinetic data of *T. brucei* TryS under the *in vivo*-like conditions described here differ significantly from published values (7). The proposed buffer system was designed to reflect the most important physicochemical features of the *T. brucei* cytosol such as environmental temperature, pH, ion composition, and total ionic strength. The intentional simplicity of the system required some compromises. Although the cytosol contains a high protein concentration, the proposed buffer does not. The validity of this simplification was supported by the fact that the activity of TryS was independent of the presence of macromolecular crowding agents, as was found previously for yeast glycolytic enzymes (22).

Because the substrates of TryS contain a number of protonable groups, the pH value of the buffer was expected to play a crucial role. The intracellular pH of bloodstream *T. brucei* is between 7.0 and 7.2 (33, 34, 49, 50). A three-compartment model of *T. brucei* that takes into account separate pH values for the cytosol, mitochondrion, and endosomal/lysosomal compartments revealed a cytosolic pH close to 7.0 (33). Indeed using a buffer at pH 7.0 instead of pH 8.0 affected all kinetic parameters of TryS. The most significant changes were observed for the *K_m_* value for Spd and the *K_i_* value for GSH, which rose by a factor of five and three, respectively. African trypanosomes multiply in the blood of their mammalian hosts. In fact, at 37 °C, *T. brucei* TryS displayed a nearly 3-fold higher activity compared with 25 °C.

For *T. cruzi*, *T. brucei*, and *C. fasciculata* TrySs, amidase activities of <1–5% compared with the respective synthetase activities have been reported (3, 7, 9). *T. brucei* TryS hydrolyzed Gsp with a *k*_cat_ of 5.1 s^−1^ and a *K_m_* value of 5.6 mm. Thus, at millimolar Gsp concentrations, the enzyme displays significant amidase activity, whereas under physiological conditions of ≤70 μm Gsp in *T. brucei* (for a review, see Ref. 51), the reaction probably does not play any significant role. The addition of ATP did not only decrease the amidase activity but even stimulated formation of T(SH)_2_. Thus, our results support the conclusion that the amidase activity in the intact parasite as well as in the kinetic analysis is negligible. Recently, a TryS double-knock-out *T. brucei* cell line that expresses an amidase-dead mutant of the enzyme was generated. These parasites proliferate *in vitro* and are capable to infect mice, although less efficiently than wild-type cells, which indicates that the amidase function is not required for viability (13).

TryS from different trypanosomatid species has been biochemically characterized (3, 7, 9, 11), but the catalytic mechanism is still not completely understood. Therefore, we decided to measure a full kinetic profile of *T. brucei* TryS under *in vivo*-like conditions and to integrate the data in a single computational model. The data and the model support a rapid equilibrium ter-reactant mechanism where all three substrates need to be bound before catalysis starts (Fig. 6). A similar mechanism has been proposed for related ATP-dependent peptide ligases such as *C. fasciculata* GspS (52, 53) and *T. brucei* γ-glutamylcysteine synthetase (54). However, for *C. fasciculata* TryS the initial formation of a quaternary complex between the enzyme and the substrates has been ruled out (3). Instead, a ping-pong mechanism has been suggested where GSH and Mg-ATP react first to generate a phosphorylated GSH intermediate that subsequently reacts with Gsp and produces T(SH)_2_. This mechanism was based on the finding of parallel lines in the reciprocal plots when Gsp was varied in the presence of saturating ATP and different fixed GSH concentrations that were below both the *K_m_* and *K_i_* values (3). For *T. brucei* TryS we analyzed a much broader range of GSH concentrations. When increasing the GSH concentration from 40 μm to 2 mm, the lines did not follow the expected order and were only partially parallel. Clearly our proposed catalytic mechanism does not conflict with an activation of GSH to glutathionyl phosphate as has been proposed for *E. coli* GspS (55); it only implies that formation of this intermediate most likely requires the prior binding of all substrates to the enzyme.

TryS catalyzes the production of both Gsp and T(SH)_2_, and different possible mechanisms about how the two reactions may be connected have been discussed (3). The crystal structure of *L. major* TryS together with molecular modeling approaches shows that the synthetase active site is a triangular shaped cavity that accommodates the three substrates roughly at the vertices of the triangle (47). There is no evidence for a second active site, and Gsp as substrate is postulated to bind to the same site as Spd (47). This is corroborated by recent docking and molecular dynamics simulations that identified the putative binding site for the glutathione moiety of Gsp (48). As reported previously for the *T. cruzi* enzyme (10), the reaction of *T. brucei* TryS with GSH and Spd did not immediately yield T(SH)_2_. Instead we observed an initial buildup of Gsp that exceeded the enzyme concentration by a factor of >100. This is clear experimental evidence that the newly formed Gsp has to leave the active site and then binds again as substrate in a different orientation suited for the second reaction. The fact that our model fits the complete dataset only when we explicitly represent Gsp generated in the first reaction in an orientation that does not allow further catalysis (E_Q in Fig. 6) further corroborates this conclusion.

Substrate inhibition of TryS by GSH is a known phenomenon. We found *K_i_* values for GSH that are well above the *K_m_* values. It could, therefore, be envisioned that at low concentrations, GSH binds almost exclusively at its substrate binding site, whereas at higher concentrations it acts also as an inhibitor. As shown here, T(SH)_2_ is also a powerful inhibitor of *T. brucei* TryS and affects the inhibition by GSH. In the model, T(SH)_2_ displayed a dual effect. There was significant binding of T(SH)_2_ both to the free enzyme in its production site (ER in Fig. 6) and to the activated enzyme XQ. Including binding to the activated complexes was essential for a good fit of the data, in particular to describe the alleviation of GSH inhibition by T(SH)_2_.

The very low *K_D_* value for binding of T(SH)_2_ to the free enzyme seems to conflict with its apparent *K_i_* values. But three aspects preclude a direct comparison; (*a*) T(SH)_2_ also inhibits by binding to the activated enzyme, (*b*) other substrates present in the reaction compete for the free enzyme and, probably as a result of this, (*c*) in steady state the concentration of free enzyme is very low, which tends to diminish the actual ER concentration.

In *E. coli* GspS, GSH cannot only bind at its catalytically competent site but was also found to partially occupy the Spd site (55). There it forms a mixed disulfide with a (non-conserved) cysteine residue. This binding site is, however, most probably an accidental trap as it was only observed in the absence of Spd (55). A molecular dynamic simulation on *L. major* TryS predicts that free GSH could also bind at the Gsp binding pocket, which would likely impair the binding of Gsp and Spd (48). Our kinetic data are not in accordance with a competitive inhibition *versus* Spd and Gsp, as increased concentrations of either polyamine substrate enhanced the inhibition by GSH instead of reducing it. Alternative models where inhibitory GSH and T(SH)_2_ bind before ATP hydrolysis could not fit the data. Hence, our model suggests that both T(SH)_2_ and GSH can act as uncompetitive inhibitors which interact with the activated enzyme complexes (XC and XQ in Fig. 6). It is thus tempting to speculate that the GSH/T(SH)_2_ inhibitory site becomes only accessible after a structural rearrangement of the enzyme due to the phosphorylation of GSH during catalysis. Large conformational changes upon binding of the substrates have been proposed for *C. fasciculata* TryS as well as *E. coli* GspS (3, 6). To summarize, our data suggest TryS is inhibited by T(SH)_2_ remaining bound at its product site, and by GSH and T(SH)_2_, sequestering the activated enzyme in unproductive complexes. The precise location of the inhibitory site will require novel structural information.

We emphasize that despite the impressive model performance, not all parameters are fully constrained by the data. The forward and reverse rate constants of binding processes were sometimes mutually dependent, implying that we can estimate the dissociation constant but not the individual rate constants. The model topology, however, is tightly constrained by the data. Various alternative mechanisms could be excluded as the corresponding models did not fit the entire dataset. For instance, an early model version with phosphorylation of GSH before binding of Spd or Gsp did not accommodate the Gsp and Spd datasets with a single parameter set. The final model captured the entire steady-state kinetics and was able to predict the product profile of HPLC experiments, which were not used in the model parameterization.

We propose that the *in vivo*-like buffer conditions developed here are adopted by others working on bloodstream form *T. brucei*. This should facilitate the standardization of data and allow the integration of kinetic data into a single computational model describing the parasite metabolism under cellular conditions. The inhibition of TryS by both GSH and T(SH)_2_ at physiological concentrations suggests that the enzyme is tightly regulated *in vivo*.

## Footnotes

5 K. Diederich, C. Hiller, and R. L. Krauth-Siegel, unpublished work.

## Footnotes

4 The abbreviations used are:

T(SH)_2_: trypanothione
TryS: trypanothione synthetase
Spd: spermidine
Gsp: glutathionylspermidine
GspS: glutathionylspermidine synthetase.
